# Synergistic lower blepharoplasty and midface lift: The role of multi-directional thread fixation

**DOI:** 10.1016/j.jpra.2026.05.006

**Published:** 2026-05-24

**Authors:** Han Earl Lee, Kyu-Ho Yi

**Affiliations:** a Opening Plastic Surgery Clinic, Seoul, Republic of Korea; b You and I Clinic, Seoul, Republic of Korea

**Keywords:** Lower Blepharoplasty, Multi-directional thread, Lifting thread, Midface lift, PDO threads, Facial rejuvenation, Minimally invasive surgery

## Abstract

**Background:**

The periorbital region and midface age as a continuous anatomic unit. Standard approaches addressing these regions separately may overlook their functional linkage. We describe a combined transcutaneous lower blepharoplasty and midface lift using multi-directional barbed polydioxanone (PDO) threads via a shared subciliary incision.

**Methods:**

This preliminary prospective consecutive case series was conducted at a single plastic surgery clinic between October 2022 and May 2023 to evaluate the early clinical outcomes and safety of combined transcutaneous lower blepharoplasty with multi-directional PDO thread midface lift. The study included 84 patients undergoing combined lower blepharoplasty and midface suspension using Sihler Thread® (multi-directional barbed PDO). Standardized pre- and postoperative photographs (4 months) were evaluated by three blinded physician evaluators. Outcomes included perceived age (pragmatic early aesthetic endpoint) and supportive validated/commonly used scales (Global Aesthetic Improvement Scale [GAIS] and a standardized infraorbital/tear-trough grading tool (Allergan Infraorbital Hollows Scale [AIHS]).

**Results:**

Mean perceived age decreased from 57.0 ± 7.5 years preoperatively to 50.0 ± 6.6 years at 4 months (mean reduction 7 years; *p* < 0.001; 95% CI 6.2–7.8). The combined approach added approximately 5–7 min and required no additional incisions. No major complications (hematoma, infection, permanent nerve injury, thread extrusion/migration) were observed.

**Conclusions:**

Combined transcutaneous lower blepharoplasty with multi-directional PDO thread midface lift via a shared incision is technically feasible and associated with early (4-month) improvement. Because perceived age is subjective and follow-up is short, results should be interpreted as preliminary; controlled comparative studies with longer follow-up and objective grading are warranted.

## Introduction

The contemporary landscape of aesthetic medicine has undergone fundamental transformation, driven by heightened societal awareness and demand for interventions targeting the visible signs of ageing.^1^^,^^2^ This paradigm shift has precipitated an exponential increase in patient requests for facial rejuvenation procedures. Individuals increasingly seek sophisticated solutions addressing the multifaceted manifestations of chronological and photoageing, compelling practitioners to expand their therapeutic repertoire across both traditional surgical interventions and innovative non-invasive modalities.^3^

The midface, defined anatomically from the lower eyelid to the nasolabial fold, is notably complex to rejuvenate effectively.^4^, ^5^, ^6^ Characteristic age-related changes in this zone include volumetric depletion, gravitational descent of the malar fat pad, and consequent development of tear trough deformities with midface ptosis.^4^ These interconnected alterations collectively generate an aged appearance that frequently proves refractory to isolated interventions.

Conventional surgical approaches to midface rejuvenation, including standard midface lifts, superficial musculoaponeurotic system (SMAS) procedures, and endoscopic techniques, typically necessitate extensive dissection, prolonged recovery periods, and carry inherent risks associated with invasive surgery.^6^^,^^7^^,^^12^ Conversely, non-surgical alternatives such as dermal fillers, laser therapies, and thread lifts offer reduced morbidity and downtime, yet often yield less dramatic or durable outcomes.^5^^,^^8^

The advent of advanced multi-directional thread technology, particularly polydioxanone (PDO) sutures featuring specialised bidirectional barb configurations, has revolutionised minimally invasive facial rejuvenation.^8^^,^^9^^,^^18^ These biocompatible, absorbable threads deliver immediate mechanical lifting through barb-mediated tissue anchorage while concurrently stimulating collagen neogenesis via controlled inflammatory responses, thereby conferring both immediate and long-term aesthetic benefits.^9^, ^10^, ^11^

**Fig. 1:**
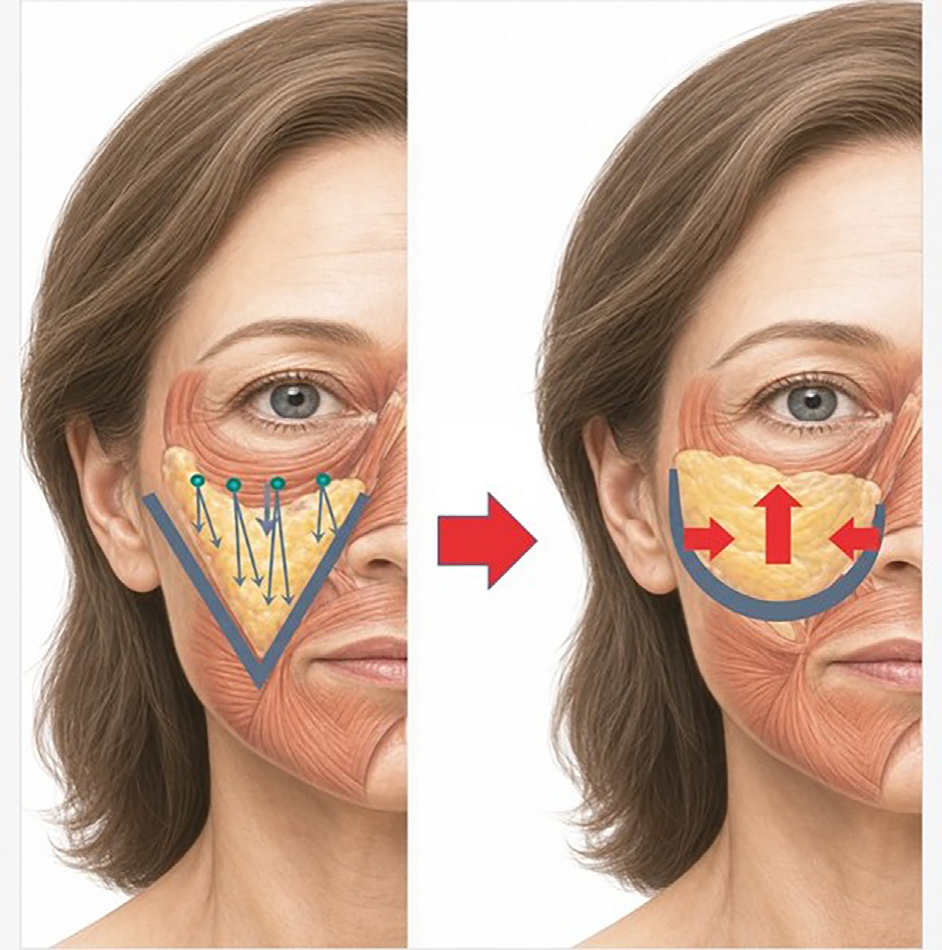
Schematic diagram illustrating the malar fat pad descent driving midface ageing. (a) Aged state: Ptotic 'V-shaped' contour caused by inferomedial descent of the malar fat pad (blue arrows indicates primary descent vector). (b) Youthful state: Characteristic 'U-shaped' contour with supported malar fat pad position.

Midface ageing is characterised by the descent of the malar fat pad, which results in the transformation from a youthful 'U-shaped' contour to a ptotic 'V-shaped' configuration (Fig. 1).^16^^,^^17^ To address this specific anatomical change, the present case series investigates a novel combined approach: integrating transcutaneous lower blepharoplasty with concurrent midface suspension using Sihler Thread® (Sihler Inc., Seoul, Republic of Korea) multi-directional PDO threads. This single-intervention technique targets the periorbital-midface continuum, aiming to correct the V-shape at its source. The synergistic combination seeks to optimise aesthetic results while reducing surgical trauma and operative burden. The primary aim of this study was to evaluate early (4-month) rejuvenation outcomes following combined transcutaneous lower blepharoplasty and multi-directional PDO thread midface lift, assessed using perceived age and complication rates.

## Methods

### Study design and population

This preliminary prospective consecutive case series was conducted at a single aesthetic surgery clinic between October 2022 and May 2023 to evaluate the early clinical outcomes and safety of combined transcutaneous lower blepharoplasty with multi-directional PDO thread midface lift. All 84 cases represented consecutive patients meeting inclusion criteria.

All participants provided written informed consent for the procedure and for the use of de-identified clinical photographs for research and publication.

Patient Selection.

Eighty-four consecutive patients were included in the study, comprising 64 females (76.2%) and 20 males (23.8%). The mean patient age was 56 years (range: 42–71 years). All patients presented with clinical evidence of lower eyelid ageing changes (including lower eyelid bags and tear trough deformity) and midface ptosis. Patients were required to have realistic expectations regarding surgical outcomes. Inclusion criteria encompassed patients aged 40–75 years with clinically evident lower eyelid bags, tear trough deformity, and midface descent.

This age range was selected as a pragmatic clinical guideline, as patients younger than 40 years often do not demonstrate sufficient midface descent to benefit from thread-assisted lifting, whereas patients older than 75 years may require more extensive surgical rejuvenation. However, we acknowledge that facial aging is a heterogeneous process, and individual variations may exist outside this age range.

Exclusion criteria included active infection, bleeding disorders, unrealistic expectations, and previous midface surgery within 12 months.

### Surgical Technique

All procedures were performed by a single experienced operator under local anesthesia with conscious sedation. The operator had >15 years of clinical experience in aesthetic periocular and midface procedures. The operator’s primary clinical specialty/training is stated in the author information and can be provided upon request.

The combined technique involved two distinct but complementary components:

#### Lower blepharoplasty

A standard transcutaneous lower blepharoplasty was performed through a subciliary incision, allowing access to the orbital fat compartments and lower eyelid structures. A skin–muscle flap was elevated routinely to expose the orbital septum and fat compartments. Selective release of the arcus marginalis and orbicularis retaining ligament was performed when indicated to facilitate fat repositioning and allow entry into the prezygomatic space for thread insertion. Following appropriate fat removal or repositioning and conservative skin excision, this incision was utilised for the concurrent midface lift procedure. In selected patients, the septal/orbicularis support and superior repositioning of lower lid tissues may reduce the need for routine canthopexy; however, canthopexy/canthoplasty remains indicated based on individual lid laxity and surgeon judgment.

#### Multi-directional thread midface lift

The midface lift component utilised Sihler Thread (multi-directional barbed PDO) threads, characterised by their multi-directional barb design that provides Y.

#### Yenhanced tissue engagement and lifting capacity (supplementary video 1)

A skin–muscle flap was elevated to expose the orbital septum and facilitate access to the prezygomatic space.

• Thread Insertion: Pairs of multi-directional PDO threads were introduced through the subciliary incision into the prezygomatic space, engaging the zygomatico-cutaneous ligament complex and adjacent fibrous septa rather than the mobile malar fat pad. Threads were directed superomedially, vertically, and superolaterally according to a predefined vector plan to achieve a reproducible lifting pattern.
• Thread Fixation: Each paired thread received a buried fixation tie within the deep fascial layer to enhance longevity while minimizing palpability. No palpable knots were detected during follow-up.
• Multiple Vector Approach: A total of four thread passes (two paired passes per side) were performed to create a three-dimensional lifting network along the superomedial, vertical, and superolateral vectors. This standardized multi-vector strategy ensured consistency and reproducibility across patients. The entire midface lift procedure was performed through the existing subciliary incision without the need for additional access points.

### Outcome assessment

Clinical photography was performed preoperatively and at 4-month follow-up using standardized lighting, camera distance, and patient positioning (frontal and 45-degree oblique views) with neutral facial expression. Three independent physician evaluators, blinded to the temporal sequence of photographs, assessed outcomes from randomized image sets.

Perceived age assessment was used as a pragmatic early postoperative aesthetic endpoint. Three independent blinded physician evaluators were asked to estimate the patient’s age based on standardized preoperative and postoperative photographs presented in randomized order. Each evaluator provided an independent age estimate, and the final perceived age was calculated as the mean value of the three assessments for each time point.

To address the inherent subjectivity of perceived age, we additionally included commonly used and standardized assessment tools: (1) the Global Aesthetic Improvement Scale (GAIS), a 5-point scale ranging from “worse” to “very much improved,” and (2) the Allergan Infraorbital Hollows Scale (AIHS), a validated photonumeric scale used to grade infraorbital hollow severity. These scales were applied consistently at baseline and at 4-month follow-up by the same blinded evaluators.

The 4-month follow-up was selected as an early time point when postoperative edema and scar maturation are typically stabilized enough for standardized photographic comparison, while still reflecting “early-stage” outcomes; longer-term follow-up is necessary to evaluate durability and the full remodeling effect of PDO threads.

The AIHS was selected because it is a commonly used photonumeric grading tool for infraorbital hollows/tear-trough severity, facilitating standardized baseline-to-follow-up comparison in routine clinical photography.

### Statistical analysis

Descriptive statistics including means and standard deviations were calculated for demographic and outcome variables. Normality of paired data was confirmed using the Shapiro–Wilk test prior to applying parametric paired t-testing. Paired *t*-tests were utilised to compare pre- and postoperative perceived ages. Statistical significance was defined as *p* < 0.05. All analyses were performed using SPSS version 28.0.

## Result

### Patient demographics

The study cohort comprised 84 patients with a mean age was 56.0 ± 7.2 years. The gender distribution reflected the typical demographic profile of aesthetic surgery patients, with a predominance of female participants (76.2%).

### Primary outcome measures

**Fig. 2:**
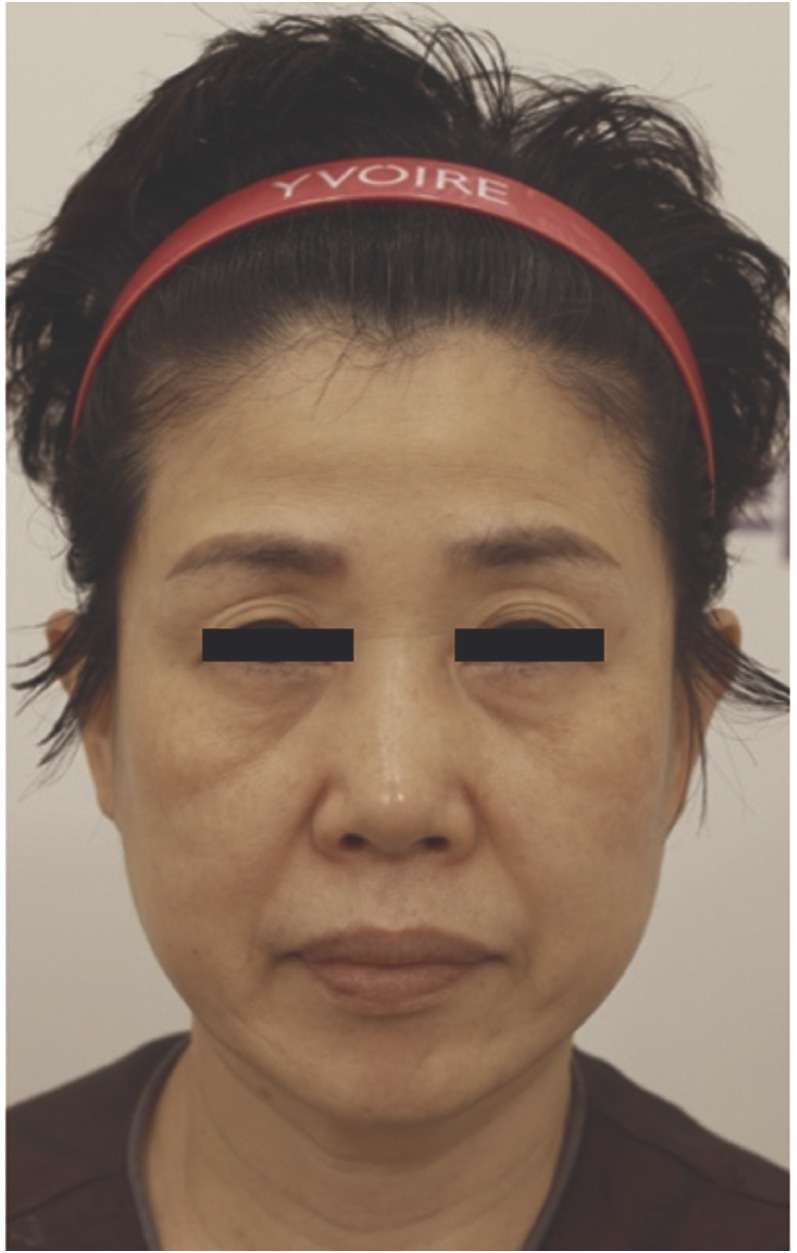

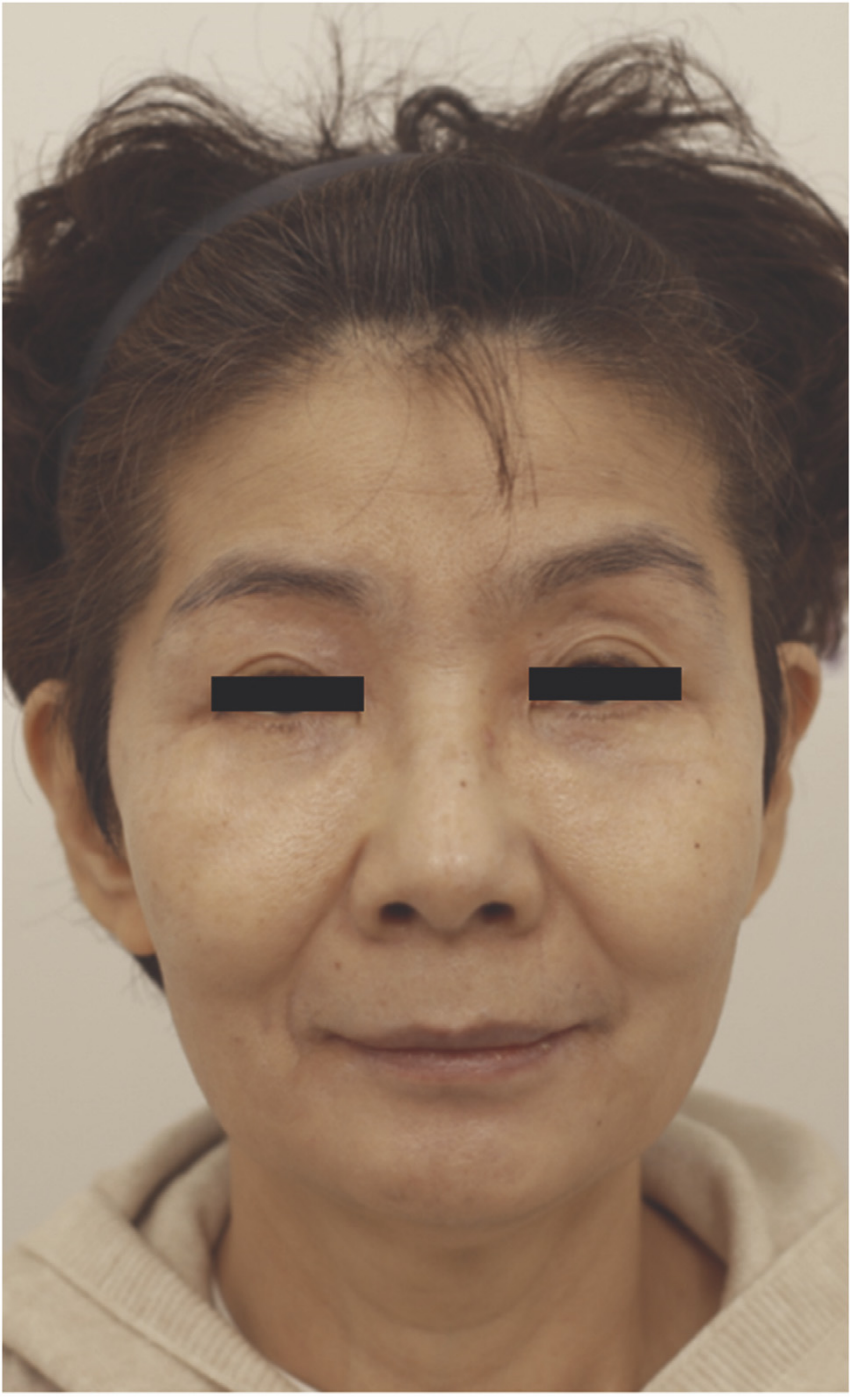
Representative 55-year-old female patient. (A) Preoperative frontal and 45-degree views (perceived age: 54 years). (B) Four-month postoperative frontal and 45-degree views following combined transcutaneous lower blepharoplasty with PDO thread midface suspension (perceived age: 48 years).

The primary outcome measure, perceived age reduction, demonstrated statistically significant improvement across the entire cohort (Fig. 2). The mean preoperative perceived age was 57.0 ± 7.5 years and decreased to 50.0 ± 6.6 years at 4 months postoperatively. This represents a mean reduction of 7 years (*p* < 0.001; 95% CI: 6.2–7.8).

(Table 1). Supportive assessments using GAIS and AIHS demonstrated consistent directional improvement across the cohort, as summarized in Table 1, with blinded evaluators generally rating postoperative appearance as improved on GAIS and on standardized infraorbital/tear-trough grading at 4 months. Detailed numerical distributions will be reported in a subsequent larger comparative study.

**Table 1:** Summary of primary and supportive outcome measures. *GAIS (Global Aesthetic Improvement Scale) and AIHS (Allergan Infraorbital Hollows Scale) were assessed by three blinded physician evaluators using standardized photographic comparisons. As this is a preliminary study, detailed numerical distributions were not included but demonstrated consistent directional improvement across the cohort.*

Outcome Measure	Preoperative	Postoperative (4 months)	Mean Change	p-value
Perceived Age (years)	57.0 ± 7.5	50.0 ± 6.6	−7.0	<0.001
GAIS (Global Aesthetic Improvement Scale)	Not applicable	Improved*	Not applicable	Not applicable
AIHS (Infraorbital Hollow Severity Scale)	Baseline grading performed	Improved*	Not applicable	Not applicable

### Operative considerations

The additional midface lift procedure required minimal additional operative time, typically adding 5–7 min to the overall procedure duration. No additional incisions were necessary, as the entire lifting procedure was performed through the existing subciliary approach.

### Clinical outcomes

Representative cases demonstrated visible early improvement in midface contour and perceived age, with outcomes appearing natural across varying anatomical presentations.

### Complications and adverse events

No major complications such as hematoma, infection, or permanent nerve injury were observed in the study cohort. Minor adverse events included temporary bruising and swelling in 22 patients (26.2%), which resolved within 2–3 weeks without intervention. No thread-related complications such as extrusion, migration, or palpable knots were encountered during the follow-up period. No patients reported palpable knots or irregularities during the follow-up period, corroborating the effectiveness of deep fascial placement described in the surgical technique.

## Discussion

This case series observed a mean reduction in perceived age of 7 years following the combined transcutaneous lower blepharoplasty and multi-directional Sihler Thread midface lift procedure. As perceived age represents an early, subjective aesthetic metric, this reduction should be interpreted as an initial postoperative observation rather than a definitive long-term outcome. Published reports of isolated lower blepharoplasty typically describe perceived age reductions of approximately 3–5 years; therefore, the greater degree of observed improvement in this cohort suggests a potential additive effect attributable to the combined approach. However, without a control group comparing isolated lower blepharoplasty, thread lift alone, or the combined technique, direct attribution of this difference remains speculative.^20^

The rationale for combining these procedures stems from the anatomical continuity between the periorbital and midface regions, where common age-related changes—including infraorbital fat pseudoherniation, ligamentous attenuation, malar descent, and tear trough formation—frequently coexist.^13^, ^14^, ^15^, ^16^, ^17^, ^19^, ^21^, ^22^ Addressing these units simultaneously through a shared incision aims to produce a more integrated correction. The combined approach specifically targets the morphological transition from a youthful convex ("U-shaped") to an aged concave ("V-shaped") malar contour. Multi-directional PDO threads contribute immediate mechanical lifting via barb anchorage and may stimulate localized collagen deposition during biodegradation, potentially enhancing early structural support.^9^^,^^10^ It must be noted, however, that the biological remodeling effects of PDO threads extend well beyond the 4-month evaluation period, and thus cannot be fully assessed within the present follow-up window.

The 4-month assessment was intentionally chosen as an early postoperative checkpoint after resolution of most postoperative swelling and stabilization of the lid–cheek contour, allowing standardized photographic comparison while minimizing confounding by acute recovery changes. We acknowledge that this early window cannot address long-term durability; extending follow-up (e.g., 12 months and beyond) is necessary to evaluate persistence of lift and the later-phase collagen remodeling effects of PDO threads.

Long-term follow-up (e.g., 12 months or longer) is required to evaluate the durability of the lifting effect and the full extent of collagen remodeling associated with PDO threads.

Regarding the interval between case accrual (ending May 2023) and submission, additional time was required for data verification, standardized photographic curation, and completion of blinded evaluations prior to manuscript preparation and submission.

Utilizing the existing lower blepharoplasty incision for access to the prezygomatic space minimizes additional surgical trauma, reduces scar burden, and enhances operative efficiency.^12^^,^^16^ The multi-directional barb configuration of the threads improves tissue engagement compared with monodirectional variants, while the multi-vector application addresses the three-dimensional nature of midface ptosis. The combination procedure added only 5–7 min to operative time, aligning with the increasing patient preference for time-efficient interventions with minimal downtime.

While PDO thread lifting has shown benefit in isolated facial contouring, its concurrent use with surgical lower blepharoplasty has been less thoroughly documented.^8^^,^^18^

Recent studies have also reported favorable outcomes using combined lower blepharoplasty and PDO thread-assisted midface lifting, demonstrating improved aesthetic results and patient satisfaction compared to isolated procedures.^23^

Conceptually, our technique aligns with prior principles of eyelid–cheek rejuvenation described in septal reset and composite approaches, where restoring the lid–cheek junction and providing vertical support are emphasized. Classic descriptions by Hamra highlight the role of septal reset in improving the youthful eyelid–cheek complex and providing a firmer undersurface support compared with fat transposition alone. These concepts support our emphasis on stabilizing the lower lid–midface transition while minimizing additional access morbidity. The early improvements observed in this cohort support the hypothesis that thread-mediated midface suspension may augment the rejuvenative effect of lower blepharoplasty. Nevertheless, such an interpretation requires caution, as synergistic outcomes cannot be confirmed without comparative or randomized studies designed to isolate the specific contribution of the threads.

Several limitations constrain interpretation and should be considered when evaluating these findings, which should be regarded as preliminary. First, as a non-comparative case series, the absence of a control group (e.g., blepharoplasty alone) prevents causal attribution and direct comparison. Second, perceived age is inherently subjective; therefore, GAIS and standardized infraorbital/tear-trough grading were incorporated as supportive measures, but fully objective morphometrics (e.g., 3D surface imaging/volumetry) were not performed. Third, the 4-month follow-up reflects early postoperative change and cannot determine long-term durability or late remodeling effects of PDO threads. Fourth, severe tear trough deformity may be difficult to correct without adjunctive volumization (e.g., fat grafting or filler); thus, our approach may have limited applicability in cases requiring significant volume restoration. These limitations underscore the need for controlled comparative studies with longer follow-up and objective endpoints.

Future research should include controlled comparative designs (blepharoplasty alone vs combined technique), longer follow-up (>12 months), patient-reported outcome measures, and objective grading/morphometric imaging (e.g., standardized infraorbital/tear-trough scales and, when available, 3D surface imaging/volumetry) to quantify durability and comparative efficacy.

## Conclusion

In this preliminary prospective consecutive case series, combined transcutaneous lower blepharoplasty and multi-directional PDO thread midface suspension via a shared subciliary incision was technically feasible and associated with early (4-month) improvement in perceived age and clinical appearance without major complications. Because the primary endpoint (perceived age) is inherently subjective and follow-up was limited to an early postoperative time point, these findings should be interpreted as preliminary; controlled comparative studies with longer follow-up and validated objective grading are needed to determine durability and comparative efficacy.

## Ethical approval

This study was conducted accordance of Declaration of Helsinki.

## Author contributions

Conceptualization: Han Earl Lee; Kyu-Ho Yi, Writing-Original Draft Preparation: Han Earl Lee; Kyu-Ho Yi, Writing-Review & Editing: Han Earl Lee; Kyu-Ho Yi, Visualization: Han Earl Lee; Kyu-Ho Yi, Supervision: Kyu-Ho Yi.

## Author disclosure

All authors have reviewed and approved the article for submission.

## Funding

The authors received no financial support for the research, authorship, and publication of this article. The products utilized in this study were donated by the injectors for the purposes of this study.

## Declaration of competing interest

None declared.

## Appendix Supplementary materials

Supplementary Video 1: Surgical demonstration of Sihler Thread midface lift performed through a single subciliary incision under conscious sedation. Image, video 1 dummy alt text
